# Supplementary material for: A Likelihood Approach for Real-Time Calibration of Stochastic Compartmental Epidemic Models
Source: PLoS Comput Biol. 2017 Jan 17;13(1):e1005257. doi: 10.1371/journal.pcbi.1005257 (PMC5240920; doi:10.1371/journal.pcbi.1005257)
Supplement: S1 File — (TAR.GZ) [file pcbi.1005257.s014.tar.gz › HSPH_Online-SI-Revision/EnKF07/n1-mild/n1-mild_EnKF2_table8.pdf]

1	newly 3 weeks spec	9.	32.52	{1., 118.}	312.889	0.520613	newly 3 weeks cumul	15.	128.12	{12., 300.}	763.067	0.658306	newly 5 weeks spec	11.	19.37	{0., 60.}	192.273	0.923526	newly 5 weeks cumul	33.	172.57	{15., 550.}	443.667	0.51495	newly 47 weeks cumul	75.	221.12	{15., 890.}	240.187	0.444916
2	newly 3 weeks spec	1.	18.77	{0., 50.}	1805.	0.908337	newly 3 weeks cumul	7.	177.21	{14., 759.}	2437.	0.933312	newly 5 weeks spec	1.	5.49	{0., 7.}	549.	0.785883	newly 5 weeks cumul	9.	192.37	{16., 803.}	2043.	0.879177	newly 43 weeks cumul	9.	205.6	{16., 815.}	2188.89	0.912866
3	newly 3 weeks spec	7.	18.55	{0., 33.}	232.714	0.600896	newly 3 weeks cumul	17.	133.91	{17., 312.}	696.765	0.63039	newly 5 weeks spec	1.	5.19	{0., 7.}	503.	0.740145	newly 5 weeks cumul	22.	148.9	{18., 477.}	588.364	0.578801	newly 52 weeks cumul	22.	156.52	{18., 546.}	622.727	0.595707
4	newly 3 weeks spec	14.	9.94	{0., 19.}	95.7143	1.03569	newly 3 weeks cumul	32.	90.9	{16., 113.}	207.375	0.343071	newly 5 weeks spec	6.	5.6	{0., 9.}	132.667	1.17931	newly 5 weeks cumul	48.	103.31	{16., 132.}	151.479	0.318451	newly 46 weeks cumul	263.	118.93	{16., 217.}	85.4259	0.722843
5	newly 3 weeks spec	5.	7.84	{0., 12.}	149.6	0.737822	newly 3 weeks cumul	17.	67.	{8., 67.}	320.353	0.418988	newly 5 weeks spec	1.	1.72	{0., 5.}	186.	0.758323	newly 5 weeks cumul	20.	72.53	{8., 77.}	292.55	0.410961	newly 48 weeks cumul	23.	74.54	{9., 84.}	258.348	0.400165
6	newly 3 weeks spec	3.	6.9	{1., 11.}	179.333	0.447339	newly 3 weeks cumul	20.	60.78	{11., 51.}	222.9	0.335623	newly 5 weeks spec	7.	2.38	{0., 4.}	93.1429	1.14174	newly 5 weeks cumul	35.	66.73	{13., 57.}	127.8	0.273291	newly 52 weeks cumul	116.	75.96	{13., 63.}	94.7931	0.615216
7	newly 3 weeks spec	5.	20.74	{0., 80.}	420.8	1.10837	newly 3 weeks cumul	17.	98.99	{6., 321.}	501.941	0.576309	newly 5 weeks spec	6.	14.08	{0., 37.}	268.	1.2672	newly 5 weeks cumul	25.	130.73	{7., 507.}	448.2	0.532058	newly 51 weeks cumul	45.	167.41	{9., 655.}	318.822	0.501242
8	newly 3 weeks spec	13.	12.11	{0., 19.}	123.462	1.15363	newly 3 weeks cumul	40.	134.28	{10., 432.}	281.75	0.455771	newly 5 weeks spec	15.	5.46	{0., 8.}	103.733	1.52266	newly 5 weeks cumul	63.	147.59	{12., 445.}	195.127	0.453367	newly 43 weeks cumul	262.	158.58	{12., 445.}	94.2824	0.776739
9	newly 3 weeks spec	0.1	4.71	{0., 8.}	4718.	0.700867	newly 3 weeks cumul	7.	93.6	{4., 92.}	1253.43	0.741381	newly 5 weeks spec	0.1	2.77	{0., 2.}	2836.	0.275311	newly 5 weeks cumul	7.	99.48	{4., 92.}	1336.86	0.763278	newly 42 weeks cumul	7.	110.04	{4., 94.}	1487.43	0.784426
10	newly 3 weeks spec	0.1	15.19	{0., 27.}	15 128.	1.58605	newly 3 weeks cumul	1.	64.94	{14., 92.}	6394.	1.6025	newly 5 weeks spec	0.1	11.57	{0., 15.}	11 550.	1.0902	newly 5 weeks cumul	1.	89.65	{14., 103.}	8865.	1.69191	newly 43 weeks cumul	1.	138.56	{15., 208.}	13 756.	1.77406
11	newly 3 weeks spec	14.	7.78	{0., 16.}	87.7143	1.05873	newly 3 weeks cumul	36.	109.97	{10., 179.}	234.639	0.40556	newly 5 weeks spec	13.	2.29	{0., 7.}	86.8462	1.57344	newly 5 weeks cumul	55.	116.13	{10., 244.}	154.782	0.375488	newly 42 weeks cumul	283.	119.38	{10., 284.}	82.6926	0.770026
12	newly 3 weeks spec	13.	14.25	{0., 22.}	127.769	0.884204	newly 3 weeks cumul	24.	136.88	{13., 230.}	488.083	0.503214	newly 5 weeks spec	5.	4.7	{0., 6.}	142.	1.15306	newly 5 weeks cumul	44.	149.18	{13., 364.}	267.955	0.380948	newly 43 weeks cumul	158.	164.25	{15., 364.}	114.259	0.562202
13	newly 3 weeks spec	2.	4.83	{0., 13.}	260.5	0.904878	newly 3 weeks cumul	5.	63.67	{6., 134.}	1182.6	0.685618	newly 5 weeks spec	0.1	0.59	{0., 1.}	654.	0.230157	newly 5 weeks cumul	5.	66.13	{6., 138.}	1231.8	0.70416	newly 44 weeks cumul	5.	66.66	{6., 138.}	1242.4	0.708519
14	newly 3 weeks spec	0.1	4.44	{0., 8.}	4444.	0.768555	newly 3 weeks cumul	3.	55.49	{9., 64.}	1752.33	0.997068	newly 5 weeks spec	0.1	1.56	{0., 2.}	1606.	0.35813	newly 5 weeks cumul	3.	59.42	{9., 70.}	1883.33	1.01842	newly 48 weeks cumul	3.	61.51	{9., 75.}	1953.	1.0276
15	newly 3 weeks spec	14.	1.75	{0., 4.}	92.5	1.67497	newly 3 weeks cumul	34.	77.09	{9., 123.}	185.5	0.399312	newly 5 weeks spec	7.	0.22	{0., 0.}	99.7143	1.79573	newly 5 weeks cumul	49.	77.79	{9., 138.}	143.694	0.471255	newly 48 weeks cumul	104.	78.06	{9., 138.}	103.712	0.686571
16	newly 3 weeks spec	6.	8.11	{0., 8.}	175.5	1.10026	newly 3 weeks cumul	34.	55.15	{4., 42.}	174.265	0.57893	newly 5 weeks spec	9.	2.41	{0., 3.}	107.889	1.66517	newly 5 weeks cumul	52.	60.96	{4., 67.}	148.923	0.709195	newly 46 weeks cumul	164.	64.64	{4., 99.}	104.671	1.11177
17	newly 3 weeks spec	12.	4.84	{0., 11.}	106.167	1.55949	newly 3 weeks cumul	46.	57.24	{2., 89.}	155.087	0.767732	newly 5 weeks spec	14.	1.35	{0., 2.}	97.5	1.91227	newly 5 weeks cumul	71.	61.05	{2., 135.}	129.592	0.899312	newly 49 weeks cumul	150.	66.57	{2., 139.}	104.313	1.14972
18	newly 3 weeks spec	9.	8.89	{0., 25.}	121.222	0.991575	newly 3 weeks cumul	36.	98.99	{12., 219.}	200.917	0.416678	newly 5 weeks spec	12.	1.92	{0., 6.}	89.5	1.55578	newly 5 weeks cumul	56.	104.64	{13., 220.}	127.393	0.368414	newly 42 weeks cumul	105.	107.11	{17., 250.}	78.3905	0.411866
19	newly 3 weeks spec	9.	26.07	{1., 67.}	239.444	0.551097	newly 3 weeks cumul	31.	153.04	{14., 393.}	415.548	0.510526	newly 5 weeks spec	2.	10.82	{0., 32.}	497.	0.716225	newly 5 weeks cumul	38.	179.22	{17., 520.}	394.421	0.513078	newly 49 weeks cumul	38.	209.59	{19., 583.}	470.605	0.550688
20	newly 3 weeks spec	2.	8.9	{0., 13.}	436.	0.870933	newly 3 weeks cumul	25.	88.63	{5., 256.}	292.92	0.45469	newly 5 weeks spec	0.1	3.63	{0., 4.}	3674.	0.43925	newly 5 weeks cumul	27.	97.69	{5., 256.}	300.111	0.459565	newly 46 weeks cumul	27.	104.03	{5., 256.}	322.778	0.469877
21	newly 3 weeks spec	2.	18.88	{4., 29.}	852.	0.844464	newly 3 weeks cumul	9.	106.59	{31., 156.}	1084.33	0.912012	newly 5 weeks spec	1.	8.61	{0., 15.}	791.	0.828775	newly 5 weeks cumul	10.	127.85	{40., 175.}	1178.5	0.958452	newly 46 weeks cumul	10.	151.84	{49., 244.}	1418.4	1.03258
22	newly 3 weeks spec	11.	4.49	{0., 9.}	110.818	1.71254	newly 3 weeks cumul	38.	98.67	{8., 114.}	201.447	0.375602	newly 5 weeks spec	8.	1.61	{0., 1.}	104.875	1.74345	newly 5 weeks cumul	55.	102.96	{8., 118.}	147.309	0.387276	newly 48 weeks cumul	274.	109.68	{8., 145.}	90.9562	0.867596
23	newly 3 weeks spec	4.	18.31	{0., 17.}	481.25	1.11679	newly 3 weeks cumul	24.	88.74	{10., 134.}	294.5	0.3873	newly 5 weeks spec	9.	9.52	{0., 6.}	176.222	1.62562	newly 5 weeks cumul	45.	111.14	{11., 170.}	202.089	0.373699	newly 43 weeks cumul	68.	137.39	{13., 367.}	186.75	0.467535
24	newly 3 weeks spec	9.	22.05	{0., 110.}	253.667	0.88204	newly 3 weeks cumul	31.	132.29	{9., 405.}	358.677	0.438405	newly 5 weeks spec	8.	7.86	{0., 19.}	149.5	1.32507	newly 5 weeks cumul	48.	152.35	{10., 433.}	265.063	0.419539	newly 45 weeks cumul	112.	172.98	{10., 608.}	160.661	0.608865
25	newly 3 weeks spec	8.	1.61	{0., 7.}	92.375	1.53627	newly 3 weeks cumul	29.	83.87	{7., 110.}	232.31	0.441556	newly 5 weeks spec	12.	0.22	{0., 0.}	98.1667	1.96362	newly 5 weeks cumul	48.	84.53	{7., 114.}	141.896	0.441209	newly 42 weeks cumul	155.	85.2	{7., 114.}	92.2581	0.767539
26	newly 3 weeks spec	3.	13.26	{0., 19.}	422.	0.840949	newly 3 weeks cumul	7.	123.49	{10., 250.}	1670.43	0.906966	newly 5 weeks spec	0.1	4.68	{0., 7.}	4696.	0.670137	newly 5 weeks cumul	11.	135.65	{12., 257.}	1143.18	0.780925	newly 45 weeks cumul	11.	145.16	{12., 294.}	1229.64	0.803175
27	newly 3 weeks spec	13.	15.04	{0., 37.}	154.615	1.29598	newly 3 weeks cumul	30.	77.19	{6., 154.}	213.767	0.413297	newly 5 weeks spec	5.	6.11	{0., 16.}	165.4	1.20648	newly 5 weeks cumul	42.	92.53	{6., 201.}	190.786	0.450856	newly 42 weeks cumul	51.	102.8	{6., 245.}	181.882	0.492081
28	newly 3 weeks spec	5.	26.11	{0., 94.}	532.2	1.05682	newly 3 weeks cumul	18.	112.93	{7., 487.}	556.944	0.535971	newly 5 weeks spec	9.	11.46	{0., 50.}	184.222	1.508	newly 5 weeks cumul	31.	142.83	{7., 714.}	410.355	0.518411	newly 48 weeks cumul	190.	159.24	{7., 779.}	120.568	0.893484
29	newly 3 weeks spec	0.1	6.49	{0., 14.}	6452.	1.09523	newly 3 weeks cumul	5.	105.52	{6., 213.}	2018.8	0.923323	newly 5 weeks spec	0.1	1.01	{0., 2.}	1070.	0.284574	newly 5 weeks cumul	5.	108.92	{7., 247.}	2086.8	0.940486	newly 45 weeks cumul	5.	109.87	{7., 247.}	2105.8	0.945135
30	newly 3 weeks spec	8.	20.31	{0., 35.}	243.875	0.754861	newly 3 weeks cumul	27.	117.49	{8., 454.}	364.556	0.46699	newly 5 weeks spec	5.	3.07	{0., 8.}	93.	1.01088	newly 5 weeks cumul	40.	129.03	{8., 476.}	260.025	0.422189	newly 49 weeks cumul	44.	133.31	{8., 478.}	242.614	0.425466
31	newly 3 weeks spec	0.1	16.77	{0., 26.}	16 714.	1.49899	newly 3 weeks cumul	0.1	115.59	{10., 184.}	115 494.	2.71129	newly 5 weeks spec	0.1	4.47	{0., 12.}	4480.	0.774541	newly 5 weeks cumul	0.1	128.37	{10., 191.}	128 274.	2.75308	newly 47 weeks cumul	0.1	135.76	{10., 276.}	135 664.	2.77777
32	newly 3 weeks spec	17.	15.23	{0., 43.}	119.471	0.972879	newly 3 weeks cumul	43.	119.62	{8., 418.}	217.767	0.423894	newly 5 weeks spec	10.	4.37	{0., 14.}	103.7	1.45604	newly 5 weeks cumul	62.	131.59	{9., 439.}	163.435	0.404756	newly 45 weeks cumul	224.	136.88	{10., 444.}	94.3214	0.73668
33	newly 3 weeks spec	3.	24.37	{0., 71.}	742.333	0.743674	newly 3 weeks cumul	15.	189.33	{11., 631.}	1174.87	0.736299	newly 5 weeks spec	1.	10.31	{0., 20.}	991.	0.819428	newly 5 weeks cumul	19.	214.23	{12., 663.}	1041.42	0.723453	newly 46 weeks cumul	20.	243.	{16., 744.}	1126.7	0.768749
34	newly 3 weeks spec	9.	29.54	{0., 104.}	318.222	0.95288	newly 3 weeks cumul	32.	137.15	{5., 568.}	376.344	0.525382	newly 5 weeks spec	6.	13.52	{0., 46.}	236.	0.97481	newly 5 weeks cumul	42.	171.22	{6., 669.}	358.762	0.521794	newly 45 weeks cumul	93.	205.7	{9., 719.}	208.645	0.594899
35	newly 3 weeks spec	10.	24.52	{1., 69.}	209.4	0.571854	newly 3 weeks cumul	37.	131.05	{15., 349.}	280.892	0.427545	newly 5 weeks spec	4.	6.93	{0., 15.}	151.75	0.731662	newly 5 weeks cumul	48.	150.64	{20., 384.}	239.792	0.401241	newly 47 weeks cumul	53.	182.63	{23., 664.}	270.057	0.437617
36	newly 3 weeks spec	0.1	22.94	{0., 75.}	22 872.	1.70032	newly 3 weeks cumul	0.1	174.96	{20., 493.}	174 860.	2.80976	newly 5 weeks spec	0.1	10.04	{0., 31.}	10 006.	1.13445	newly 5 weeks cumul	0.1	199.56	{22., 727.}	199 460.	2.87588	newly 49 weeks cumul	0.1	223.84	{22., 807.}	223 740.	2.92957
37	newly 3 weeks spec	5.	21.61	{0., 34.}	383.8	0.596086	newly 3 weeks cumul	27.	121.16	{21., 328.}	362.	0.483451	newly 5 weeks spec	7.	7.97	{0., 8.}	159.571	1.12371	newly 5 weeks cumul	39.	142.35	{23., 339.}	284.692	0.421073	newly 45 weeks cumul	50.	158.7	{25., 431.}	243.4	0.387351
38	newly 3 weeks spec	2.	15.25	{0., 22.}	723.5	0.793978	newly 3 weeks cumul	11.	127.76	{14., 421.}	1069.09	0.74011	newly 5 weeks spec	0.1	3.26	{0., 6.}	3266.	0.688818	newly 5 weeks cumul	12.	138.25	{15., 435.}	1059.42	0.741059	newly 49 weeks cumul	12.	148.01	{15., 439.}	1140.75	0.76038
39	newly 3 weeks spec	4.	24.51	{3., 44.}	528.25	0.564806	newly 3 weeks cumul	11.	93.41	{17., 133.}	753.727	0.736426	newly 5 weeks spec	4.	15.62	{0., 33.}	340.	0.607795	newly 5 weeks cumul	27.	128.49	{20., 204.}	387.741	0.508827	newly 47 weeks cumul	33.	165.27	{23., 298.}	413.848	0.52038
40	newly 3 weeks spec	0.1	7.36	{0., 21.}	7394.	0.628324	newly 3 weeks cumul	1.	130.69	{4., 469.}	12 979.	1.54893	newly 5 weeks spec	0.1	0.63	{0., 1.}	706.	0.185312	newly 5 weeks cumul	1.	133.27	{4., 470.}	13 237.	1.55729	newly 50 weeks cumul	1.	133.76	{4., 470.}	13 286.	1.56019
41	newly 3 weeks spec	3.	16.83	{0., 42.}	503.667	0.596596	newly 3 weeks cumul	11.	74.31	{7., 156.}	592.273	0.585715	newly 5 weeks spec	0.1	8.9	{0., 24.}	8878.	1.05553	newly 5 weeks cumul	11.	95.53	{7., 247.}	782.818	0.645804	newly 51 weeks cumul	11.	119.32	{7., 434.}	998.909	0.710248
42	newly 3 weeks spec	0.1	16.85	{0., 17.}	16 778.	1.54998	newly 3 weeks cumul	0.1	148.52	{18., 367.}	148 420.	2.81566	newly 5 weeks spec	0.1	4.52	{0., 7.}	4528.	0.710173	newly 5 weeks cumul	0.1	161.64	{20., 391.}	161 540.	2.85346	newly 50 weeks cumul	0.1	171.29	{20., 399.}	171 190.	2.87469
43	newly 3 weeks spec	0.1	9.03	{0., 11.}	8992.	1.21758	newly 3 weeks cumul	5.	117.16	{16., 242.}	2243.6	0.971639	newly 5 weeks spec	0.1	3.87	{0., 6.}	3882.	0.679081	newly 5 weeks cumul	5.	126.3	{17., 340.}	2426.	1.01258	newly 52 weeks cumul	5.	137.83	{17., 359.}	2656.6	1.05353
44	newly 3 weeks spec	3.	9.13	{0., 13.}	274.333	0.669965	newly 3 weeks cumul	3.	67.52	{6., 91.}	2154.67	1.07007	newly 5 weeks spec	0.1	2.92	{0., 5.}	2936.	0.618127	newly 5 weeks cumul	3.	75.5	{7., 117.}	2420.67	1.11481	newly 44 weeks cumul	3.	84.9	{7., 138.}	2734.	1.14778
45	newly 3 weeks spec	7.	11.46	{0., 27.}	158.571	0.894449	newly 3 weeks cumul	21.	89.35	{17., 135.}	336.524	0.474196	newly 5 weeks spec	2.	4.79	{0., 13.}	257.5	0.962377	newly 5 weeks cumul	25.	101.03	{17., 200.}	317.48	0.460811	newly 42 weeks cumul	30.	117.73	{17., 327.}	308.7	0.450773
46	newly 3 weeks spec	13.	12.05	{0., 21.}	92.8462	0.745343	newly 3 weeks cumul	34.	64.98	{14., 112.}	118.176	0.329283	newly 5 weeks spec	14.	6.43	{0., 11.}	96.6429	1.24723	newly 5 weeks cumul	70.	80.02	{14., 125.}	74.2286	0.332306	newly 49 weeks cumul	231.	106.65	{15., 168.}	78.4978	0.664601
47	newly 3 weeks spec	8.	25.25	{0., 112.}	355.625	1.33014	newly 3 weeks cumul	29.	165.02	{5., 714.}	518.483	0.561883	newly 5 weeks spec	9.	8.26	{0., 16.}	157.778	1.58643	newly 5 weeks cumul	47.	188.29	{5., 821.}	375.298	0.611673	newly 47 weeks cumul	220.	199.24	{5., 872.}	129.464	0.983468
48	newly 3 weeks spec	9.	14.07	{0., 49.}	179.222	1.10739	newly 3 weeks cumul	23.	93.92	{10., 206.}	339.826	0.464926	newly 5 weeks spec	13.	5.68	{0., 20.}	100.923	1.51279	newly 5 weeks cumul	45.	108.09	{10., 257.}	199.578	0.457397	newly 42 weeks cumul	52.	122.01	{10., 329.}	200.712	0.483775
49	newly 3 weeks spec	14.	22.15	{1., 66.}	152.5	0.628358	newly 3 weeks cumul	26.	161.2	{16., 690.}	534.231	0.525667	newly 5 weeks spec	13.	6.36	{0., 12.}	101.846	1.29975	newly 5 weeks cumul	51.	179.2	{22., 693.}	278.941	0.386811	newly 46 weeks cumul	150.	188.87	{22., 732.}	121.167	0.484218
50	newly 3 weeks spec	9.	11.77	{0., 17.}	113.444	0.572298	newly 3 weeks cumul	30.	66.26	{9., 85.}	149.267	0.38578	newly 5 weeks spec	4.	5.31	{0., 7.}	146.25	0.845062	newly 5 weeks cumul	48.	79.5	{9., 100.}	104.792	0.339574	newly 43 weeks cumul	70.	93.47	{9., 106.}	93.8143	0.368773
